# Supplementary material for: WordCommentsAnalyzer: A windows software tool for qualitative research
Source: F1000Res. 2018 Sep 4;7:536. Originally published 2018 May 3. [Version 2] doi: 10.12688/f1000research.14819.2 (PMC6137411; doi:10.12688/f1000research.14819.2)
Supplement: Supplementary file 3 [file f1000research-7-17614-s0002.tgz › b2a79496-4ada-47c4-9682-a99ce867836d.zip!/Eight Iranian Health Journals/Journal of Health System Research _23.docx]

© Journal of Health System Research . This work is licensed under CC BY-NC 4.0 (https://creativecommons.org/licenses/by-nc/4.0/).

Volume & Issue: دوره 9، شماره 9؛ 1392

Title: مروری بر تاثیر تغییرات آب و هوایی بر کیفیت آب و غذاهای دریایی

http://hsr.mui.ac.ir/index.php/jhsr/article/view/1112

تغییرات آب و هوایی در سراسر جهان در حال حاضر یک واقعیت است که تاثیرات مختلفی بر محیط زیست و سلامت انسان و موجودات دارد. در مورد تاثیر تغییرات آب و هوایی بر سیستم مواد غذایی، به خصوص غذاهای دریایی از مزرعه تا سفره اطلاعات کمی وجود دارد. هدف از این مطالعه مروری، بررسی بنیادین اثرات تغییرات آب و هوایی بر ایمنی غذاهای دریایی و پیامدهای آن بر سلامت انسان است. پیش‌بینی می‌شود تغییرات آب و هوایی باعث افزایش آلودگی‌های شیمیایی و بیولوژیکی مانند فلزات سمی، باقیمانده‌های مواد شیمیایی آلی، جلبک‌های سمی و سموم آنها و پاتوژن‌ها شود که تهدیدی جدی بر سلامت و امنیت غذاهای دریایی داشته و از طریق زنجیره غذایی سلامت انسان را به خطر می‌اندازد که نیاز به توجه جدی در این زمینه است.

Title: مجـلات پزشکی و چاپ تبلیغات

http://hsr.mui.ac.ir/index.php/jhsr/article/view/966

امروزه مجلات علوم پزشکی در تمامی دنیا، نقش مهم و تاثیرگذار خود را در سلامت جوامع انسانی به اثبات رسانده‌اند. با این وجود، یک سؤال اساسی که همیشه در خصوص این مجلات مطرح می‌گردد این است که آیا مجلات علوم پزشکی باید اقدام به چاپ تبلیغات نمایند یا خیر؟ هدف از نگارش مقاله مروری حاضر، بحث پیرامون جنبه‌های بسیار مهمی است که در پاسخ به این سؤال، باید مورد توجه قرار گیرند. در پایان سال 1391 موتور جستجوی پاب مد با بررسی واژگان  medical, journal, advertising در عنوان مقاله مورد جستجو قرار گرفت. در مرحله بعد، با تغییر در شیوه جستجو و هم‌چنین با بهره‌گیری از منابع مورد استفاده در مقالات به دست آمده، نهایت دقت به عمل آمد تا مرتبط‌ترین و به‌روزترین مقالات، مورد استفاده این مطالعه مروری قرار گیرند. برخی از مجلات علوم پزشکی برای تامین منابع مالی خود، عمدتا وابسته به تبلیغاتی هستند که توسط شرکت‌های بزرگ دارویی طراحی می‌گردد. این در حالی است که تاثیرات سوء برخی از این قبیل تبلیغات می‌تواند به عنوان یک خطر بزرگ، نه تنها سلامت جامعه بلکه روند تصمیم‌گیری‌های سردبیران را نیز تحت شعاع خود قرار دهد. مجلات علوم پزشکی باید تا جای ممکن از چاپ هرگونه تبلیغی خودداری کرده و یا چاپ انواع گوناگونی از تبلیغات را، آن‌هم بر اساس اصول صحیح اطلاع‌رسانی، مبنای کار خود قرار دهند..

Title: بررسی ارتباط افسردگي و خودکارآمدی در رفتارهای ارتقاء دهنده سلامتی بیماران ديابتي نوع2 شهرستان های فريدن و چادگان، سال 1391

http://hsr.mui.ac.ir/index.php/jhsr/article/view/947

مقدمه: فاکتورهای فردی، رفتاری، روانی و اجتماعی در بروز اختلالات روانی می‌تواند موثر باشد. هدف مطالعه حاضر بررسی شیوع و شدت افسردگی و تعیین ارتباط آن با خودکارآمدی در بیماران دیابتیک نوع 2 شهرستان‌های فریدن و چادگان می‌باشد. روش‌ها: نوع مطالعه توصيفي – تحليلي و از نوع مقطعی، که بر روی 403 بیمار در دو شهرستان و به روش تصادفی سیستماتیک با رعایت معیارهای ورود و خروج انجام شد. ابزار جمع‌آوری اطلاعات، پرشسنامه، شامل 9سؤال در خصوص مشخصات دموگرافیک و تست استاندارد 21 سؤالی افسردگی بك و 6 سؤال در خصوص خودکارآمدی بود. تجزیه و تحلیل داده‌ها به وسیله نرم‌افزارSPSS  نسخه 18و با استفاده از آزمون‌های آماری تی مستقل، کای اسکوئر، من- ویتنی، کروسکال والیس و اسپیرمن در سطح معني‌داري 05/0انجام گرديد. يافته‌ها: 160نفر(7/39%) بیماران فاقد افسردگی، 109نفر (27%) دارای افسردگی خفیف، 94 نفر (3/23%) افسردگی متوسط و 40نفر (9/9%) دارای افسردگی شدید می‌باشند. شیوع افسردگی در زنان بیشتر از مردان و فاقد تفاوت معني‌داري بود         (27/.=P) بین شیوع و شدت افسردگی و متغیرهای سن بالاتر، محل سکونت، سطح تحصیلات پایین، مدت زمان ابتلا به بیماری دیابت، شیوع عوارض، ابتلا همزمان به بیماری‌های مزمن دیگر و خودکارآمدی پایین رابطه معنی‌داری دیده شد (05/0 P<). نتیجه‌گیری: به نظر می‌رسد متغیرهای فردی، رفتاری، اجتماعی و فرهنگی و دسترسی همگانی به خدمات، تاثیر مهمی در کاهش شدت افسردگی در این بیماران را داشته باشند. بنابراین پیشنهاد می‌شود بحث مهم عدالت در سلامت بایستی به طور جدی و دقیق انجام گیرد .

Title: بررسی رابطه بین استرین گرمایی و بهره وری نیروی انسانی در یک صنعت مونتاژ خودرو

http://hsr.mui.ac.ir/index.php/jhsr/article/view/961

مقدمه: افراد در بسیاری از مشاغل در معرض استرس‌های حرارتی قرار دارند. مواجهه با گرما و ایجاد تنش گرمایی در فرد، علاوه بر تاثیر منفی بر روی سلامتی فرد، بهره‌وری و راندمان او را تحت تاثیر قرار می‌دهد و به همین دلیل سبب کاهش کمیت و کیفیت تولید در سازمان می‌گردد. لذا هدف این مطالعه بررسی رابطه بین استرین گرمایی و بهره‌وری نیروی انسانی در صنعت مونتاژ خودرو بود. روش‌ها: این مطالعه مقطعی در دو فصل تابستان و زمستان 1390 بر روی 181 نفر از کارگران صنعت مونتاژ خودرو انجام گرفت. جهت بررسی استرین گرمایی از دو شاخص دمای تر گویسان و نمره استرین گرمایی استفاده شد. همچنین جهت بررسی وضعیت بهره‌وری نیروی انسانی از مقیاس 26 سؤالی بهره‌وری نیروی انسانی هرسی و گلداسمیت استفاده گردید. جهت بررسی ارتباط شاخص‌های دمای تر گویسان و نمره استرین گرمایی با بهره‌وری و ریزمقیاس‌های تشکیل‌دهنده آن از آزمون ضریب همبستگی پیرسون استفاده گردید. یافته‌ها: بیشترین و کمترین مقدار شاخص‌های دمای تر گویسان و شاخص نمره استرین گرمایی در فصل تابستان، به ترتیب برای واحد مخصوص و سوله انبار به دست آمد. در فصل زمستان بیشترین مقدار این شاخص‌ها برای واحد تولید به دست آمد و کمترین مقدار این شاخص‌ها در فصل زمستان، به ترتیب برای واحدهای بازرگانی و مخصوص به دست آمد. بیشترین مقدار بهره‌وری هم برای واحد بازرگانی (25/81) و کمترین مقدار آن برای واحد انبار (16/52) به دست آمد. نتایج حاصل از ضریب همبستگی پیرسون نشان داد که شاخص دمای تر گویسان با بهره‌وری نیروی انسانی رابطه معناداری ندارد (134/0=P). اما شاخص نمره استرین گرمایی با بهره‌وری نیروی انسانی رابطه‌ی معکوس و معناداری دارد (015/0=P) و با افزایش نمره شاخص تنش گرمایی مقدار بهره‌وری کاهش پیدا می‌کند و همچنین با کاهش نمره شاخص تنش گرمایی، مقدار بهره‌وری افزایش می‌یابد. نتیجه‌گیری: تنش گرمایی تاثیر منفی بر روی بهره‌وری نیروی انسانی دارد و با افزایش ریسک مواجهه با استرس‌های گرمایی، میزان بهره‌وری افراد کاهش پیدا می‌کند. همچنین شاخص نمره استرین گرمایی ابزار مناسبی جهت تشخیص تاثیر منفی استرین گرمایی بر روی بهره‌وری نیروی انسانی می‌باشد در حالی که شاخص دمای تر گویسان این تاثیر منفی را گزارش نکرد.

Title: برآورد ظرفیت هوازی و تعیین سطح فعالیت بدنی دانشجویان

http://hsr.mui.ac.ir/index.php/jhsr/article/view/968

مقدمه: ارتباط آشکار بین ظرفیت هوازی (vo2max) و فعالیت فیزیکی در طول تحقیقات گسترده‌ای در جهان ارایه شده است.این مطالعه با هدف برآورد ظرفیت هوازی و تعیین فعالیت فیزیکی و عوامل دموگرافیک موثر بر آن در دانشجویان پسر انجام شد. روش‌‌ها: در این مطالعه مقطعی، 68 نفر از دانشجویان پسر دانشکده بهداشت دانشگاه علوم پزشکی اصفهان که از سلامتی کامل برخوردار بودند،تصادفی انتخاب و پس از آگاهی از هدف مطالعه در تست شرکت می‌کردند.افراد مورد مطالعه به وسیله آزمون ارگومتری – پروتکل استراند مورد ارزیابی قرار گرفتند. ابزار گردآوری داده‌ها، پرسشنامه بود که مربوط به اطلاعات دموگرافیک دانشجویان بود. بررسی ارتباط بین متغیرها درج شده و vo2max با استفاده از SPSS نسخه 20 صورت گرفت. یافته‌ها: میانگین و انحراف استاندارد سن دانشجویان پسر مورد مطالعه به ترتیب برابر 39/2± 13/23 سال به دست آمد. ظرفیت هوازی در دانشجویان پسر مورد مطالعه برابر با 39/0± 23/3 لیتر بر دقیقه شد. نتایج این مطالعه نشان که بین این متغیر و فعالیت فیزیکی، سابقه ورزشی و مصرف دخانیات رابطه معنی‌داری موجود است (05/0P< ). نتیجه‌گیری: متغیرهایی همچون فعالیت فیزیکی، مصرف دخانیات و سابقه ورزشی، می‌تواند تاثیری برvo2max  داشته باشد.

Title: بررسی خصوصیات دموگرافیک مراجعه‌کنندگان به مرکز ژنتیک پزشکی اصفهان و ارتباط آن با ازدواج خویشاوندی در سال 1388

http://hsr.mui.ac.ir/index.php/jhsr/article/view/1013

مقدمه: ازدواج‌های خویشاوندی در وراثت انسان بسیار با اهمیت هستند. این الگوی ازدواج از لحاظ فرهنگی، اجتماعی و اقتصادی در بین کشورها و حتی در داخل یک کشور متفاوت است. با توجه به این مطلب که ازدواج خویشاوندی در ایران به عنوان یک الگوی ازدواج هنوز پایدار است و می‌تواند به دلایل گوناگون در آینده نیز ادامه داشته باشد، این مطالعه با هدف شناخت عوامل جمعیتی موثر بر ازدواج خویشاوندی و ارایه راهکارهای عملی و کاربردی در جهت کاهش ازدواج‌های خویشاوندی پر خطر در شهر اصفهان انجام شد. روش‌ها: روش تحقیق در این پژوهش تحلیل ثانویه داده‌های موجود در مرکز ژنتیک پزشکی اصفهان بود. جامعه آماری در این تحقیق ، کلیه زوج‌های مراجعه‌کننده به مرکز ژنتیک پزشکی اصفهان بوده‌اند. داده‌ها با بررسی پرونده‌های موجود در چهار ماهه اول سال 1388 جمع‌آوری شده‌اند. در مجموع تعداد 2091 پرونده مورد بررسی قرار گرفته است. داده‌ها با استفاده از آزمون‌های آماری مناسب و نرم افزار SPSS  نسخه 18و در سطح معنی‌داری کمتر از 05/0 تجزیه و تحلیل گردید. یافته‌ها: 4/50٪ از مراجعه‌کنندگان زن که ازدواج خويشاوندي داشته‌اند در گروه سني 20-15 سال بودند. بيشترين درصد (8/86٪) از زنان مراجعه‌کننده به این مرکز که تحصيلاتشان زير ديپلم بوده است با يکي از خويشاوندان خود ازدواج کرده‌اند. در حدود 39٪ از زنان خانه‌دار و 4/50٪ از زنان محصل يا دانشجو ازدواجشان خويشاوندي بوده است. همچنین 5/83 ٪ از مراجعه‌کنندگان زن که والدینشان ازدواج فامیلی داشته‌اند خودشان نیز ازدواج فامیلی داشته‌اند. نتیجه‌گیری: این مطالعه ارتباط معناداری بین سن ازدواج زنان و تحصیلات زنان و اشتغال زنان با ازدواج خویشاوندی را نشان داد به طوری که زنان در گروه‌های سنی پایین‌تر بیشتر ازدواجشان خویشاوندی بوده است. همچنین با افزایش تحصیلات زنان ازدواج فامیلی کاهش پیدا کرده و زنان شاغل بیشتر ازدواجشان غیرخویشاوندی بوده است.

Title: بررسی کارایی فرایند الکتروکوآگولاسیون در تصفیه شیرابه حاصل از کارخانه کمپوست اصفهان

http://hsr.mui.ac.ir/index.php/jhsr/article/view/1019

مقدمه: شیرابه کمپوست اغلب حاوی آلاینده‌های آلی، آمونیاک، فلزات سنگین، آمونیوم، کلراید، جامدات معلّق و نمک‌های معدنی و ترکیبات محلول یا معلّق و البته باکتری‌ها می‌باشد که اثرات نامطلوبی بر محیط زیست دارد. هدف از انجام این تحقیق بررسی تصفیه‌پذیری شیرابه کارخانه کمپوست اصفهان با استفاده از الکتروکوآگولاسیون است. روش‌ها: پس از انعقاد الکتریکی با استفاده از جفت الکترودهای آهن، آلومینیوم و الکترود تجاری آهن- آلومینیوم، در زمان‌های مختلف (5، 10و 20 دقیقه) و رقّت‌های 1/0 ، 025/0 و 01/0 پارامترهای EC،COD ،pH ، TSS و TDS روش استاندارد بررسی شد. پس از آنالیز داده‌ها، میانگین پارامترهای کیفی شیرابه ناشی از الکترولیز با هم مقایسه شد. یافته‌ها: کارایی حذف COD برای الکترودهای Al-Al،  Fe-Feو Fe-Al به ترتیب 36/93%، 15/95% و 02/94% و برای TSS نیز به ترتیب 3/47%، 43/37% و 86/39% در مدت زمان 20 دقیقه الکترولیز با رقّت 1/0 بود. کارایی حذف COD در رقّت 025/0 و مدت زمان تماس20 دقیقه برای الکترودهای Al-Al، Fe-Fe و Fe-Al  به ترتیب 88/97%، 08/98% و 4/98% و کارایی حذف TSS نیز به ترتیب 65/63%، 05/44% و 86/54% به دست آمد. برای رقّت 01/0 در مدت زمان تماس 20 دقیقه کارایی حذف COD برای الکترودهای Al-Al، Fe-Fe  وFe-Al  به ترتیب 28/99%، 32/99% و 41/99% و برای TSS به ترتیب 32/74%، 78/58% و 94/65% حاصل شد. نتیجه‌گیری: نتایج نشان داد که کارایی حذف پارامترها با زمان رابطه مستقیم و با pH رابطه عکس دارد. راندمان حذف COD و TSS توسط الکترودهای آهن و آلومینیوم تقریباً یکسان بود. الکترودهای آهن در کاهش COD و الکترودهای آلومینیوم در کاهش رنگ، کدورت و TSS بیشترین تأثیر را دارند. حداکثر راندمان زمانی حاصل می‌شود که از جفت الکترود آهن و آلومینیوم استفاده شود.

Title: ارائه برنامه آموزشی مبتنی بر الگوی پرسید به منظور ارتقا رفتارهای پیشگیری‌کننده از سرطان پوست در دانش‌آموزان شهر تهران

http://hsr.mui.ac.ir/index.php/jhsr/article/view/1052

مقدمه: در سرطان پوست پیشگیری اولیه بیشتر بر کاهش خطر ابتلا و کاهش عوامل خطرزا در افراد سالم تأکید دارد و از ملزومات پیشگیری اولیه، آموزش بهداشت به افراد سالم جامعه می‌باشد. هدف این مطالعه ارایه برنامه آموزشی مبتنی بر الگوی پروسید به منظور ارتقا رفتارهای پیشگیری‌کننده از سرطان پوست در دانش‌آموزان دبیرستانی شهر تهران در سال 1391 بود. روش‌ها:  این مطالعه از نوع مقطعی بوده و بر روی 450 دانش آموزان دختر و پسر مقطع دبیرستان منطقه 11 شهر تهران در سال 1391 انجام شد. روش نمونه‌گیری خوشه‌ای تصادفی شده بود. به منظور شناخت کامل‌تر از وضعیت دانش‌آموزان در جامعه مورد بررسی در رابطه با عوامل پیش‌بینی‌کننده انجام رفتارهای پیشگیری‌کننده از سرطان پوست و نیز سازماندهی مطالعه بر طبق عوامل مشخص شده در الگوی پرسید، ابزار مطالعه که یک پرسشنامه محقق ساخته شامل سازه‌های مدل پرسید بود، تدوین گردید. تهیه برنامه آموزشی با یک جستجوی گسترده متون شامل مرور علایم و نشانه‌های سرطان پوست، علل ایجاد بیماری، درمان بیماری، بار بیماری و هزینه‌های ناشی از آن، راه‌ها و روش‌های پیشگیری از سرطان پوست و برنامه‌های آموزشی تهیه شده در ارتباط با سرطان پوست و تأثیر برخی از متغیرهای روان شناختی مانند خودکارآمدی و حمایت اجتماعی بر ارتقا رفتار در دانش‌آموزان بود. سطح معنی‌داری در این مطالعه 05/0 در نظر گرفته شد. یافته‌ها: همبستگی مثبت و معنی‌داری بين رفتارهای پیشگیری‌کننده از سرطان پوست و همه متغیرهای تحت مطالعه به جز نگرش وجود داشت. همه متغیرها به جز متغیر آگاهی که با متغیرهای حمایت اجتماعی و خودکارآمدی همبستگی معنی‌دار نداشت با هم همبستگی معنی‌دار و مستقیم داشتند. تحلیل رگرسیون نشان داد که متغير هاي مستقل 43٪ از واریانس رفتارهای پیشگیری‌کننده از سرطان پوست را پیش‌بینی می‌نمایند و در این میان عوامل قادر کننده قویترین پیش بینی کننده بود. نتيجه‌گيري: بخش پرسید از الگوی پرسید-پروسید چارچوب مفهومی مناسبی را جهت طراحی برنامه آموزشی پیشگیری از سرطان پوست برای دانش‌آموزان فراهم نمود. به منظور اجرای برنامه ارایه‌شده در این مطالعه و نیز ارزشیابی آن، مطالعات بیشتری در آینده پیشنهاد می‌شود.

Title: مقایسه الگوی فعالیت عضلات اندام تحتانی قبل و بعد از فرود تک‌پا

http://hsr.mui.ac.ir/index.php/jhsr/article/view/1132

مقدمه: حرکت فرود تک پا از جمله حرکات ورزشی متداول است که احتمال آسیب‌دیدگی اندام تحتانی در آن زیاد است. با وجود این، بیومکانیک این حرکت کمتر مورد توجه قرار گرفته است، بنابراین هدف مطالعه حاضر مقایسه الگوی فعالیت عضلات اندام تحتانی در مراحل قبل و بعد از فرود تک پا بود. روش‌ها: مطالعه نیمه‌تجربی حاضر روی 13 نفر از دانشجویان مرد رشته تربیت بدنی در سال 1391 انجام شد. فعالیت الکترومایوگرافی عضلات سرینی میانی، دوسررانی، دوقلوی داخلی، نعلی و درشت‌نی قدامی همزمان با داده‌های نیروهای عکس‌العمل زمین اندازه‌گیری گردید. آزمودنی‌هاحرکت فرود تک پا را از ارتفاع 30 سانتی‌متر روی صفحه نیرو انجام دادند. حرکت فرود به دو مرحله تقسیم گردید: 100 میلی ثانیه قبل از تماس آغازین پا با زمین و 100 میلی ثانیه بعد از تماس پا با زمین (مرحله جذب نیرو). آزمون‌های آماری آنالیز واریانس و t زوجی به ترتیب برای مقایسه فعالیت عضلات در هر مرحله و بین مراحل فرود استفاده شدند. سطح معنی‌داری 05/0P≤ در نظر گرفته شد. یافته‌ها: آزمون آنالیز واریانس اختلاف معناداری را در فعالیت عضلات در هر دو مرحله نشان داد (001/0P≤). نتایج آزمون t زوجی نشان داد میزان فعالیت عضلات سرینی میانی، دو سر رانی، نعلی و درشت‌نی قدامی در مرحله جذب نیرو بیشتر از مرحله قبل از فرود بود (05/0P≤)، درحالی‌که میزان فعالیت عضله دوقلوی داخلی در مرحله قبل از فرود بیشتر از مرحله جذب نیرو بود (001/0P≤). نتیجه‌گیری: به نظر می‌رسد اندام تحتانی با فعال‌سازی عضله دوقلوی داخلی قبل از تماس پا زمین خود را برای جذب نیروها هنگام فرود آماده می‌کند. بعد از تماس با زمین نیز با توجه به نوع فرود به کار رفته در مطالعه حاضر (فرود سخت) عضلات پلنتارفلکسور نقش اصلی را در جذب نیروهای عکس‌العمل زمین در حرکت فرود تک پا بر عهده دارند.

Title: پایش پدیده مخرب لایه بندی حرارتی در دریاچه سد مخزنی اکباتان همدان

http://hsr.mui.ac.ir/index.php/jhsr/article/view/1275

مقدمه: سدها از مهم ترين سازه‌هاي تامين کننده آب آشامیدنی می‌باشند و به منظور مديريت كيفيت آب در مخازن سدها شناخت پديده‌هايي كه در آن رخ مي‌دهد، ضروری می‌باشد. از مهمترین این رخدادها، پدیده لایه بندی حرارتی می‌باشد که موجب کاهش کیفیت آب خروجی از سد می‌گردد. در این پژوهش رژیم حرارتی مخزن سد اکباتان همدان که مهمترین منبع تامین‌کننده آب قابل شرب شهر همدان می‌باشد مورد بررسی قرار گرفت. روش‌ها: در ابتدا موقعیت ایستگاه‌های نمونه‌برداری تعیین شد و در ادامه پروفیل‌های دما – عمق مخزن با اندازه‌گیری مستقیم دما در اعماق مختلف مخزن رسم گردید، در مجموع در هفت ایستگاه دما و اکسیژن محلول با فواصل 50 سانتی متری و تا عمق 25 متری از سطح آب دریاچه و در طی 12 ماه به صورت ماهیانه اندازه‌گیری شد. در نهایت نتایج به دست آمده با نمودارهای ویتزل مقایسه گردید. یافته‌ها: لایه بندی حرارتی در سد مورد نظر از اردیبهشت ماه آغاز می‌گردد و تا اوایل دی ماه به مدت 8 ماه ادامه می‌یابد و در اواسط تابستان به شدیدترین حالت خود می‌رسد، در دی ماه لایه بندی تخریب گشته و تا اواخر فروردین به مدت 4 ماه مخزن در وضعیت اختلاط کامل به سر می‌برد. لایه آلوده ترموکلاین در اکثر ماه‌هایی که آب در وضعیت لایه‌ای قرار داشت در عمق 10 متری قابل تشخیص بود. نتیجه‌گیری: باتوجه به موقعیت جغرافیایی دریاچه و تراز میانگین 1955متر، دریاچه مورد نظر جزء دریاچه‌های یک گردشی گرم با یک گردش زمستانه به مدت 4 ماه طبقه بندی می‌گردد،. بنابراین عمق برداشت در ماه‌هایی که لایه بندی وجود دارد فاکتور مهمی در کیفیت آب خروجی از مخزن سد می‌باشد.

Title: بررسی سازه‌های تئوری رفتار برنامه ریزی شده در خصوص فعالیت بدنی دانشجویان دانشگاه پیام نور نایین در سال 1391

http://hsr.mui.ac.ir/index.php/jhsr/article/view/1054

مقدمه: یکی از پیامدهای صنعتی شدن زندگی ، کاهش فعالیت بدنی در بین افراد است که یکی از مهمترین مشکلات جامعه امروزی می‌باشد؛ لذا در این پژوهش،آگاهی و سازه‌های تئوری رفتار برنامه‌ریزی شده شامل نگرش، قصد رفتاری، هنجارهای انتزاعی و کنترل رفتاری درک شده در خصوص فعالیت بدنی دانشجویان دختر دانشگاه پیام نور نائین در سال 1391 بررسی شده است. روش‌ها: در این مطالعه مقطعی 130 نفر از دانشجویان دختر به روش نمونه‌گیری تصادفی ساده انتخاب شدند. به منظور جمع آوری اطلاعات، پرسشنامه با روایی و پایایی قابل قبول، شامل سه بخش اطلاعات جمعیتی، آگاهی و سازه‌های تئوری رفتار برنامه‌ریزی شده، استفاده گردید . تجزیه و تحلیل داده‌ها به کمک نرم‌افزار 18 SPSS و آزمون‌های آماری مرتبط انجام شد. یافته‌ها: در این مطالعه130 دختر با میانگین سنی8/2±22 شرکت کرده بودند. میانگین نمرات آگاهی، نگرش، قصد، هنجارهای انتزاعی و کنترل رفتاری درک شده به ترتیب 6/14±74، 2/14± 1/81، 3/25± 3/52، 9/23±3/60 و3/ 13±1/48 بود. ارتباط معناداری بین قصد انجام فعالیت‌بدنی با آگاهی (011/0=P)، نگرش (000/0=P)، هنجارهای انتزاعی (000/0=P) و کنترل رفتاری درک شده (019/0=P)  وجود داشت.    نتیجه‌گیری: نتایج نشان داد که قصد انجام فعالیت بدنی با سازه‌های نگرش و هنجارهای انتزاعی ارتباط معناداری دارد بنابراین می توان با برگزاری جلسات آموزشی مبتنی بر تغییر نگرش مثل جلسات بحث گروهی و همچنین برگزاری جلسات آموزشی برای افراد تاثیرگذار بر فرد مثل خانواده، دوستان، همکلاسی‌ها و ..ارتقا فعالیت‌بدنی در بین دانشجویان دختر باشیم.
